# Is urinary toxicology rapid screening a useful first-line diagnostic tool for paediatric coma in the emergency department?

**DOI:** 10.3389/fped.2026.1821957

**Published:** 2026-06-05

**Authors:** Irene Malni, Luisa Zupin, Egidio Barbi, Alessandro Amaddeo, Maura Marin

**Affiliations:** 1 Department of Pediatrics, Montebelluna Hospital, Azienda ULSS 2 Marca Trevigiana, Montebelluna, Treviso, Italy; 2 Pediatric Department, Institute for Maternal and Child Health IRCCS “Burlo Garofolo”, Trieste, Italy; 3 Department of Medicine, Surgery and Health Sciences, University of Trieste, Trieste, Italy

**Keywords:** coma, paediatrics emergency care, rapid urine screening (RUS), toxic ingestion, urine toxicology screening

## Abstract

**Introduction:**

Coma in paediatric patients is a medical emergency requiring prompt identification of the underlying cause to prevent serious complications. Among the most frequent and often underrecognized causes in young children are accidental ingestions of cannabinoids and opioids, which may go unreported by caregivers either because unwitnessed or for lack of awareness of the potential risks. In this context, rapid urine screening (RUS) is a highly valuable diagnostic tool: it is inexpensive, quick, easy to perform, and sensitive to many common neurotoxic substances. In this study, we systematically reviewed the available literature on Medline regarding paediatric patients with intoxication-induced coma presenting to the emergency department.

**Methods:**

A systematic review of the literature was performed in PubMed.

**Results:**

32 case reports encompassing 57 paediatric cases were identified. Urine screening enabled identification of the toxic agent in approximately two-thirds of patients (20 RUS), helping guide early therapeutic decisions. Although qualitative and not always reflective of the severity of intoxication, RUS remains effective in the initial clinical assessment. However, RUS is not considered as a potentially useful early investigation, to be performed after glucose level and blood tests, before other procedures such as CT scan, lumbar puncture or EEG, in most paediatric guidelines.

**Discussion:**

The available epidemiological evidence of the last few years suggests that routine implementation of RUS in children under 4 years of age presenting with altered consciousness may reduce the need for invasive procedures such as lumbar puncture or computed tomography scan and support timely, targeted management.

## 1 Introduction

Coma represents a critical medical emergency, characterized by complex diagnostic and therapeutic challenges. It is a nonspecific clinical manifestation of diffuse cerebral dysfunction, secondary to structural, metabolic, or mixed aetiologies (1).

The causes of coma in the paediatric population are numerous and heterogeneous, making early and accurate diagnosis essential—not only to ensure survival but also to minimize long-term neurological sequelae. The therapeutic window for effective clinical intervention is exceedingly narrow. Paediatricians working in emergency departments and intensive care units (ICUs) frequently encounter comatose children. The incidence of non-traumatic coma is estimated at approximately 30 cases per 100,000 children per year, whereas traumatic brain injury (TBI) exhibits a substantially higher incidence, at around 670 cases per 100,000 children annually (1).

Coma represents a neurological emergency that necessitates immediate evaluation and intervention. Following Paediatric Advanced Life Support (PALS) algorithm (2), initial management focuses on the stabilization of airway, breathing, and circulation and a rapid neurological assessment. In paediatric patients with a Glasgow Coma Scale (GCS) score below 8, endotracheal intubation is indicated, and mechanical ventilation should be initiated if respiratory effort is inadequate. Adequate oxygenation must be always ensured. Vascular access should be promptly established to facilitate fluid resuscitation in cases of shock, intravenous glucose administration for hypoglycaemia, and collection of samples for diagnostic evaluation. Anticonvulsant therapy is indicated in the presence or suspicion of seizures. If there are signs of elevated intracranial pressure—such as pupillary asymmetry, abnormal posturing, or signs of cerebral herniation—urgent measures to reduce intracranial pressure are imperative. Correction of acid-base and electrolyte imbalances, along with maintenance of normothermia, is also essential (3).

In cases of coma with unclear aetiology, it is essential to broaden the diagnostic work-up to include potential toxicological causes. In this context, most available paediatric protocols for the management of coma recommend urine toxicology screening as a second-line investigation, to be performed when the initial clinical assessment fails to identify a specific cause or when there is a well-founded suspicion of poisoning by exogenous substances (4). Recent years have seen a rise in reports of intoxication-induced comas in both the medical literature and daily clinical practice, highlighting a growing concern over increased drug availability in households and the associated risk of accidental pediatric exposure. An increase in accidental exposure of children under the age of 5 to analgesic medications—including opioids—as well as to antipsychotic and antidepressant drugs, has been reported. Moreover, analgesics are also the most frequently implicated drug category in fatal poisonings in children under 5 years of age (5). These accidents may be underrecognized as they may go unreported by caregivers, either because unwitnessed or for lack of awareness of the potential risks.

So far, rapid urinary screening (RUS) is not considered as a potentially useful early investigation, to be performed after testing glucose, electrolytes, or infection markers and after stabilizing airway and circulation, before other procedures such as CT scan, lumbar puncture or EEG, in most paediatric guidelines. In this study we systematically reviewed the available evidence on paediatric patients with intoxication-induced coma at the emergency department, with a focus on aspects related on the diagnostic process.

## 2 Methods

### 2.1 Literature search and inclusion criteria

We conducted a systematic literature review of paediatric patients with intoxication-induced coma presenting to the emergency department in accordance with PRISMA (Preferred Reporting Items for Systematic Reviews and Meta-Analyses) guidelines (available at https://www.prisma-statement.org).

The search was performed by two authors (I.M. and M.M) on Medline using the following keywords: [“Poisoning” (MeSH Terms) OR “Substance-Related Disorders” (MeSH Terms)] AND [“Coma” (MeSH Terms) OR “Consciousness Disorders” (MeSH Terms)] AND [“Child” (MeSH Terms)] AND (accidental OR unwitnessed OR unexplained OR “unknown cause”) AND (child OR paediatric OR infant OR toddler OR adolescent) AND (coma OR “altered consciousness” OR unconsciousness) AND (poisoning OR intoxication OR overdose) AND (unwitnessed OR “unknown cause” OR unexplained). Only studies published between 1971 and 2023 were considered. Moreover, the references lists of the selected articles were screened to identify additional potentially eligible studies.

### 2.2 Study selection and synthesis of data

The inclusion criteria consisted of studies reporting paediatric patients under 18 years of age who presented with a GCS score <13 or with symptoms described as “coma,” “lethargy,” or “drowsiness,” in which the ingestion or exposure to the toxic substance was unintentional, whether witnessed or not. All study designs were eligible for inclusion, including case reports, case series, and observational studies.

We excluded all studies reporting patients over 18 years of age, patients with suicidal intent, patients who arrived deceased at the emergency department, and intoxications resulting from iatrogenic errors.

For each included study, the following data were extracted and recorded by two authors (I.M. and M.M.): patient age, presenting symptoms, type of substance involved, whether ingestion was witnessed, whether urine toxicology screening was performed and its contribution to the diagnosis, additional diagnostic tests conducted and their nature, use of antidote, and finally, patient outcome.

Descriptive statistics were used to synthesize the data, by using the frequency, percentage and proportion.

### 2.3 Risk of bias assessment

The risk of bias was assessed by two authors (I.M. and M.M.) following the Joanna Briggs Institute (JBI) critical appraisal tools designed for different type of studies, including case report case series, observational studies, studies (available at https://jbi.global/critical-appraisal-tools). The JBI critical appraisal suggested an overall acceptable methodological quality of the included reports. However, the overall level of evidence remains inherently limited by the observational and descriptive nature of case reports and small case series (Supplementary Table S1).

## 3 Results

The initial screening of titles and abstracts identified 48 studies. After full-text retrieval, 13 studies met the inclusion criteria and were selected. In addition, 31 studies were identified from the reference lists of these articles and 19 were selected. In total, 32 articles were included (6–37), describing 57 patients, with urine screening being performed in 41 of them.

**Figure 1:**
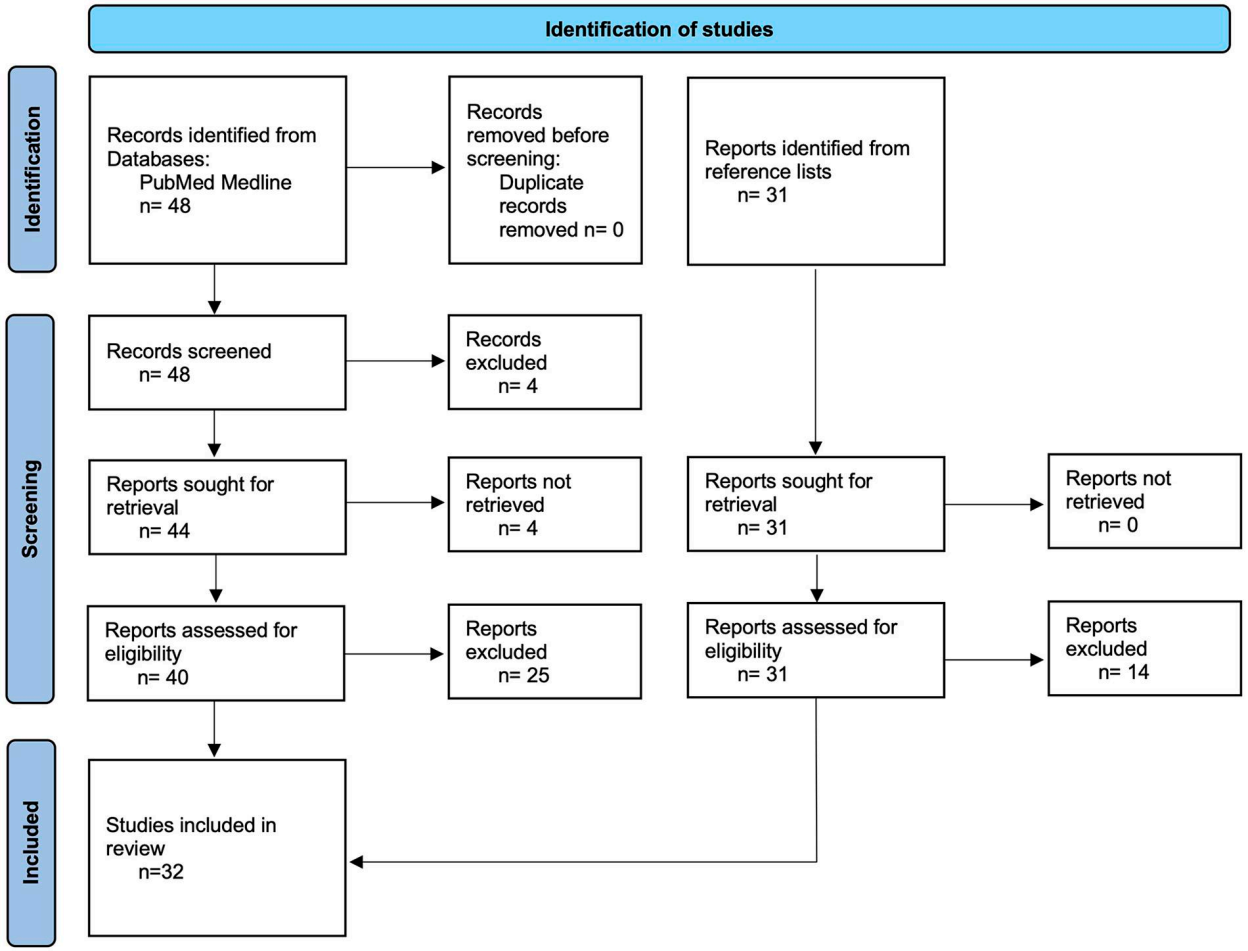
The flowchart of the study selection process.

The flow chart of the search was displayed in Figure 1.

The median age of the included patients was 37 months, with a minimum age of 8 months and a maximum of 15 years and the median age was 24 months. No significant sex-related differences were observed (27 males, 29 females), except for one case in which the patient's sex was not reported (Table 1 and Supplementary Table S2).

**Table 1:** Summary of the paediatric cases with intoxication induced coma presenting to the emergency department.

Citation	Study group	Study type	Key results	Comments	RUS panel
Mehamha et al., France (6)	Nine patients (from 11 to 25 months of age, 5 females, 4 males), presenting with at ED with various symptoms, including coma, drowsiness, hypotonia, respiratory failure, eye revulsion, seizure, conjunctival hyperemia, cries, mydriasis, muscle rigidity, vomiting, tachycardia	Case report (4)	Cannabis was detected in blood in 9/9 patients	Toxicological qualitative immunoassay was confirmed by quantitative analysis of cannabis and it metabolites	RUS included THC—KIMS (Kinetic interaction of microparticles in a solution) with a COBAS 8000 system (Roche, Bâle, Suisse)
			Cannabis was detected in urine by immunoassay and LC-MS/MS in 8/9, in 1 case the urine test was not reported no clinical consequence		
de Marcellus et al., France (8)	Male patient (48 months of age) presenting at ED with coma	Case report (4)	Baclofen was detected in blood	Urine toxicological screening was negative then baclofen ingestion was suspected, and its presence was confirmed in plasma	RUS not included baclofen
			Urine toxicological screening was negative the patient was treated with supportive therapy no clinical consequence		
Reisner et al., USA (9)	Female patient (24 months of age) presenting at ED with lethargy	Case report (4)	Morphine and hydromorphone were detected in blood	Urine toxicological test was performed at the presentation in ED, resulting in positive result for opiates, therefore during the hospital course the blood analysis confirmed the data and suggested the ingestion of sustained-release morphine (e.g., morphine sulfate)	RUS included opiates
			Opiates were detected in urine patient was treated with naloxone and surgery he was alive with disability		
Foy et al., USA (10)	Female patient (19 months of age) presenting at ED with coma, seizure	Case report (4)	Toxicological blood screening was negative as well as urine immunoassay fentanyl patch was found on patient’ skin patient was treated with naloxone and phenobarbital (for seizure) no clinical consequence	The urine screening was negative but synthetic opioids were not detected by this assay. The findings of the fentanyl patch on the skin (used by a parent) determined the cause of her symptoms	RUS not included synthetic opioids
Borzutzky et al., Chile (11)	Female patient (10 years of age) presenting at ED with lethargy, stupor, visual hallucination, hyporeflexia	Case report (4)	Toxicological blood screening was not performed clozapine was detected in urine the patient was treated with supportive therapy no clinical consequence	The patient ingested a pill for headache.	RUS not performed
				A parent had a prescription for clozapine.	
				Toxicological analysis of urine taken at ED admission identified the substance.	
Morris et al., USA (12)	Male patient (48 months of age) presenting at ED with coma	Case report (4)	Toxicological blood and urine screening were negative, the intoxication was caused by tea tree oil the patient was treated with supportive therapy no clinical consequence	The ingestion of tea tree oil was witnessed.	RUS not included Tea tree oil
				Blood and urine toxicological screening were negative	
Guidet et al., France (14)	Three patients (from 8 to 18 months of age, 3 females) presenting at ED with various symptoms, including tachycardia, drowsiness, behavioral disorder, bradypnea, mydriasis, hypotension	Case report (4)	Cannabis was detected in blood in 2/3 patients (in one patient's blood test was not reported) and in urine by immunochemical assay and HPLC-MS) in 3/3 no clinical consequence	In the three cases cannabis was firstly detected by immunochemical assay and then confirmed by quantification with HPLC-MS, in 2 cases the blood analysis further confirms the urine analysis (in 1 case blood test was not reported)	RUS included THC—[CEDIAVR, ThermoFisherVR Scientific [Waltham, MA, USA] on COBASVR 8000 RocheVR analyser [Basel, Switzerland]].
Vo et al., USA (15)	Six patients (from 7 to 15 years of age, 1 female, 5 males) presenting at ED with various symptoms, including lethargy, tachycardia, tachypnea, dizziness, dry mucosa membranes, confusion, conjunctival injection, rotory nystagmus, tachycardia, confusion, hypertension, mydriasis, abdominal pain, dizziness, nausea, nystagmus, blurry vision, horizontal nystagmus, anxiety, shortness of breath	Case report (4)	Cannabis was detected in blood in 4/6 patients (in two patients’ blood test was not reported)	Urine screening was positive in the patients analyzed, and in those cases where blood analysis was also performed, the results confirmed the urine findings	RUS included THC
			Cannabis was detected in urine by immunoassay in 5/6 patients (in one case was not reported) 4 patients were treated with supportive therapy, 1 with activated charcoal, 1 with activated charcoal and ondansetron no clinical consequence		
Feliu et al., France (16)	Female patient (16 months of age) presenting at ED with drowsiness, unable to open eyes, mydriasis	Case report (4)	Cannabis was detected in blood and urine (immunochromatography and MS quantitative analysis) no clinical consequence	Cannabis was detected in urine by immunochromatography assay and confirmed by quantitative analysis of cannabinoids and its metabolites) in urine and blood, suggesting acute oral cannabis consumption.	RUS included THC
				Moreover, the analysis of hair revealed a chronic environmental exposure to cannabinoids	
Molly et al., France (17)	Female patient (10 months of age) presenting at ED with impaired consciousness, drowsiness and restlessness, generalized hypotonia, inadequate smiles	Case report (4)	Cannabis was detected in blood and urine patient was treated with supportive therapy no clinical consequence	Cannabis was detected in both blood and urine.	RUS not specified; unclear whether it was performed prior to quantitative analysis
Amirav et al., Israel (20)	Male patient (18 months of age) presenting at ED with lethargy, ataxia	Case report (4)	Cannabis was detected in blood and urine (immunoassay and quantitative analysis) the patient was treated with supportive therapy no clinical consequence	The urine screening identified cannabis, and its presence was confirmed by urine quantitative analysis and blood quantification	RUS included THC
Bury et al., Australia (22)	Female patient (30 months of age) presenting at ED with seizure, diarrhea, vomit, jaundice	Case report (4)	Phenylbutazone was detected in blood and urine (HPLC-MS) the patient was treated with supportive therapy no clinical consequence	The patients was extremely ill for 72 h (unconsciousness and seizure), then phenylbutazone tablets were found in her vomitus and the substance was also later identified in urine and blood	RUS was not performed, generally phenylbutazone is not included in RUS
Glatstein et al., Canada (25)	Female patient (11 months of age) presenting at ED with lethargy, miotic pupils	Case report (4)	Blood analysis was not reported	Urine analysis identified methadone and its metabolites	RUS not performed
			Methadone was detected in urine patient was treated with naloxone no clinical consequence		
Lalkin et al., Canada (26)	Male patient (54 months of age) presenting at ED with lethargy, miotic pupils	Case report (4)	Methadone was detected in blood and urine (HPLC) patient was treated with naloxone no clinical consequence	Methadone was detected both in blood and in urine. Intoxication probably derived from an error in antibiotic preparation in pharmacy	RUS not performed
Riascos et al., USA (27)	Female patient (22 months of age) presenting at ED with coma	Case report (4)	Blood analysis was not reported	Urine screening identified methadone, acetaminophen and salicylates. The patient present toxic encephalopathy and died.	Case report not specified which type of urine screening was performed
			Methadone was detected in urine patient was treated with naloxone death		
Leblanc et al., France (28)	Male patient (12 months of age) presenting at ED with coma, miotic pupils	Case report (4)	Methadone was detected in blood and not detected in urine the patient was treated with naloxone no clinical consequence	The tests were initially negative. When intoxication by methadone was suspected, it was specifically search and it was found in blood.	RUS was performed, but methadone was not included in the panel
Anselmo et al., Portugal (29)	Male patient (36 months of age) presenting at ED with coma, no reaction to pain	Case report (4)	Blood analysis was negative	The urine screening was initially negative but after 36 h it tested positive for methadone	RUS was performed but resulted negative, overcorrection of the acidosis with bicarbonate led to alkaline urine and negative testing for methadone.
			Methadone was detected in urine after 36 h the patient was treated with naloxone no clinical consequence		
Tiras et al., France (31)	Three patients (from 24 to 36 months of age, 1 female, 2 males) presenting at ED with various symptoms including coma, bradypnea, miotic pupils, no reaction to pain	Case report (4)	Methadone was detected in blood (2/3) and urine (2/3, 1 LC-MS, qualitative analysis + GC-MS). IN 1 case blood and urine analysis were not reported patients were treated with naloxone 2 patients were alive with no consequence 1 patient was alive with neurologic sequelae	Medical history revealed methadone ingestion in the children.	RUS was performed in patient n.2 and resulted positive, not performed in n1. and n. 3
				In 2 cases this was confirmed by toxicological analysis; in one of them, the qualitative assay was further validated by quantitative testing	
Castro et al., Spain (32)	One male patient (24 months of age) presenting at ED with coma, miotic pupils	Case report (4)	Blood analysis was not performed	Qualitative assay revealed the presence of methadone in urine	RUS was perfomed, methadone was included in panel
			Methadone was detected in urine patients were treated with naloxone no clinical consequence		
	One female patient (24 months of age) presenting at ED with lethargy				
Schwab and Caggiano, USA (34)	One male patient (8 months of age) presenting at ED with coma, miotic pupils	Case report (4)	Blood analysis was not performed methadone was detected in urine the patient was treated with naloxone no clinical consequence	In the first child initially the screening negative. Since methadone intoxication was suspected in both children, methadone was specifically search.	RUS were negative for opiates, methadone was not included in panel. A second specimen tested specifically for methadone was positive.
	One male patient (36 months of age) presenting at ED with coma, miotic pupils				
Roland et al., Canada (35)	One male patient (9 months of age) presenting at ED with coma, miotic pupils, flaccidity	Case report (4)	In patient 1 methadone was not detected in blood but was detected in urine with immunoassay and GC	Urine screening identified the substance, and its presence was confirmed by quantitative urine analysis.	RUS not performed
	1 female patient (15 months of age) presenting at ED with apnea, coma				
			In patient 2 blood analysis was not reported, methadone was detected in urine both patients were treated with supportive therapy no clinical consequence		
				The intoxication was likely caused by an erroneous diluent used in the preparation of an antibiotic in pharmacy	
Gayle et al., Canada (36)	Female patient (14 months of age) presenting at ED with coma, difficult of breathing	Case report (4)	Blood analysis was not reported methadone was detected in urine the patient was treated with naloxone no clinical consequence	Urine and gastric aspirates were positive for methadone that derived for an erroneous antibiotic preparation	RUS not performed
Mady et al., USA (37)	One female patient (4 years of age) presenting at ED with lethargy, ataxia, confusion, diaphoretic, drooling	Case report (4)	In patient 1 clozapine was detected in blood but not detected in urine patient was treated with activated charcoal, magnesium citrate, diphenhydramine in patient 2 blood analysis was not reported and urine analysis was negative patient was treated with activated charcoal, sorbitol no clinical consequence	In patient 1 initial urine and serum toxicology screenings were initially negative, however, 5 h after ingestion, clozapine was detected in serum.	RUS performed, clozapine not detected
				In patient 2 the urine drug screening was negative, but the clozapine intoxication was suspected.	
	One male patient (21 months of age) presenting at ED with lethargy, ataxia				

In “study type” column the CEBM Levels of Evidence was reported (https://www.cebm.ox.ac.UK/).

In 33 cases, the intake of the substance was not directly observed; in 10 cases, there was a reported witness account of the toxic agent intake, while in the remaining 14 cases, such information was not documented.

Regarding clinical presentation, the mean Glasgow Coma Scale (GCS) score was 6, with a minimum of 3 and a maximum of 13; however, the GCS score was not reported in 30 cases. The level of consciousness among the selected patients ranged from drowsiness, confusion, and lethargy to a fully comatose state. Respiratory compromise was observed in 12 out of 57 cases (21%) and was described as tachypnoea, bradypnea, or apnoea. Ocular involvement—including mydriasis, miosis, or nystagmus—were reported in 20 patients (36%). Additionally, seizures occurred in 6 patients (10%).

With respect to the types of substances involved, Table 2 displays the toxic agents identified ranked in descending order of frequency. Cannabis emerged as the most frequently detected substance, followed by methadone.

**Table 2:** Distribution of identified toxic agents.

Types of substances	Frequency
Cannabis (all form)	19 (36.5%)
Methadone	17 (32.7%)
Baclofen	5 (9.6%)
Amitraz	4 (7.7%)
Tea tree oil	1 (1.9%)
Olanzapine	1 (1.9%)
Morphine	1 (1.9%)
Phenylbutazone	1 (1.9%)
Fentanyl	1 (1.9%)
Ethanol	1 (1.9%)
Carbamazepine	1 (1.9%)

Distribution of identified toxic agents.

Out of a total of 57 cases, only one fatality was reported. It involved a 22-month-old girl with methadone poisoning who arrived at the Emergency Department with a Glasgow Coma Scale (GCS) score of 3 (27). Despite the administration of naloxone, the patient did not survive and progressed to brain death. In this case, the urine toxicology screen tested positive. Two patients survived with residual disabilities (9, 31). No sequelae were observed in the remaining patients (Supplementary Table S1).

Regarding treatment, the antidote or specific treatment for intoxication was administered in 25 cases (43.8%); specifically, naloxone was given in 16 cases (28%); activated charcoal was administered in 9 cases (15%), forced alkaline diuresis in 1 case (1.7%), phenobarbital in 1 case (1.7%) (for treating seizure). All 16 cases, in which naloxone were administered, were opioid intoxication. In the remaining cases, treatment was either not reported or consisted only of supportive care, such as mechanical ventilation support, hydration, and clinical observation in a hospital setting.

Blood tests were performed in 41 out of 57 patients; in 34 of these cases (corresponding to 60% of the total), the presence of the toxic substance in the blood was confirmed.

Regarding the urine toxicology test, in 16 cases it was either not performed, or its execution was not reported in the article.

In 5 cases, the urine toxicology test was performed with negative results, including two cases of clozapine (37), one case of fentanyl (10), one case of baclofen (8) and a case of tea tree oil intoxication (12), in one more case (29) it was initially negative, and it became positive after 36 h. In the remaining 35 cases, corresponding to 85% of the total, the test detected the presence of toxic substances at the first assessment. In 20 cases qualitative assay was performed, followed by substance quantification in 14 cases. In 4 cases the urine test was a quantitative analysis, while in the remaining cases the method used was not specified. Of the 35 positive cases, cannabinoids were detected in 19, opioid in 1, clozapine in 1, NSAID in 1. Interestingly, methadone was detected in 12 cases, in one more case positivity was observed only after 36 h, and in another it was found in blood but not in urine (28).

When considering only the rapid urine method, in only 1 case the rapid urine toxicology screening was negative despite fentanyl intoxication (10), while in the remaining 19 cases, corresponding to 95% of the total, the test detected the presence of toxic substances at the first assessment, specifically cannabinoids in 17, and methadone in 2.

The cases of patients intoxicated with cannabinoids included in this review span from 2011 to 2021; it is noteworthy that nearly all these cases have been reported in case reports published after 2015.

**Figure 2:**
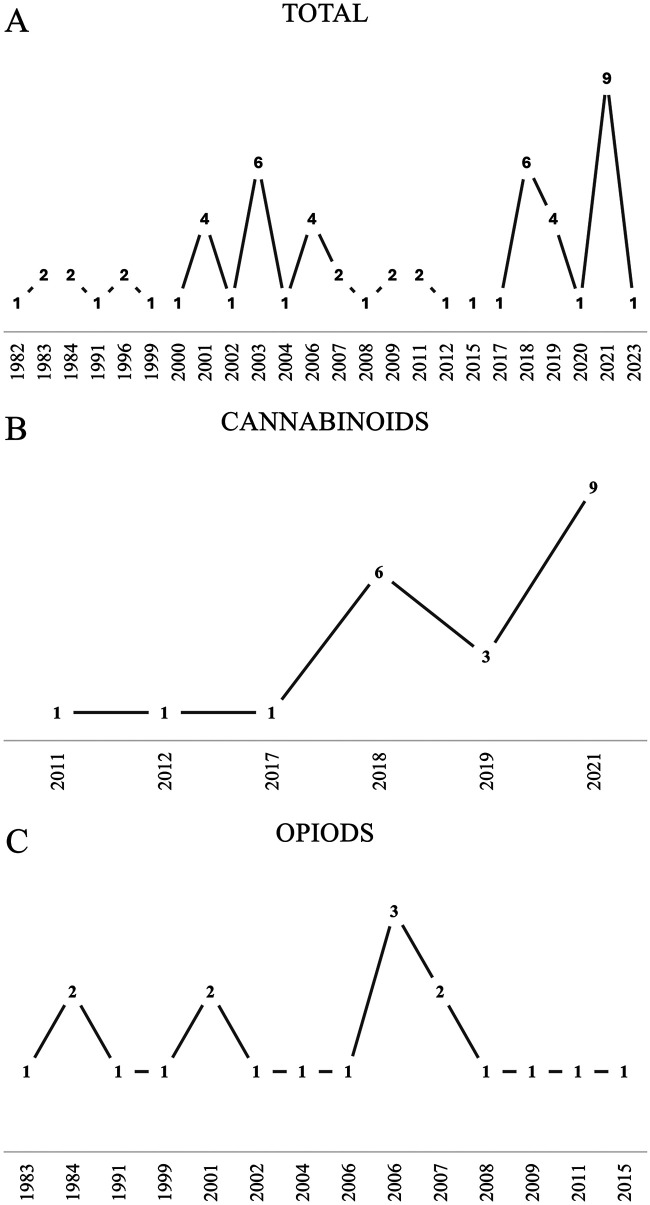
Timetables of reported cases. **(A)** All cases; **(B)** cannabinoids; **(C)** opioids.

The timetables of the cases reported were presented in Figure 2.

**Figure 3:**
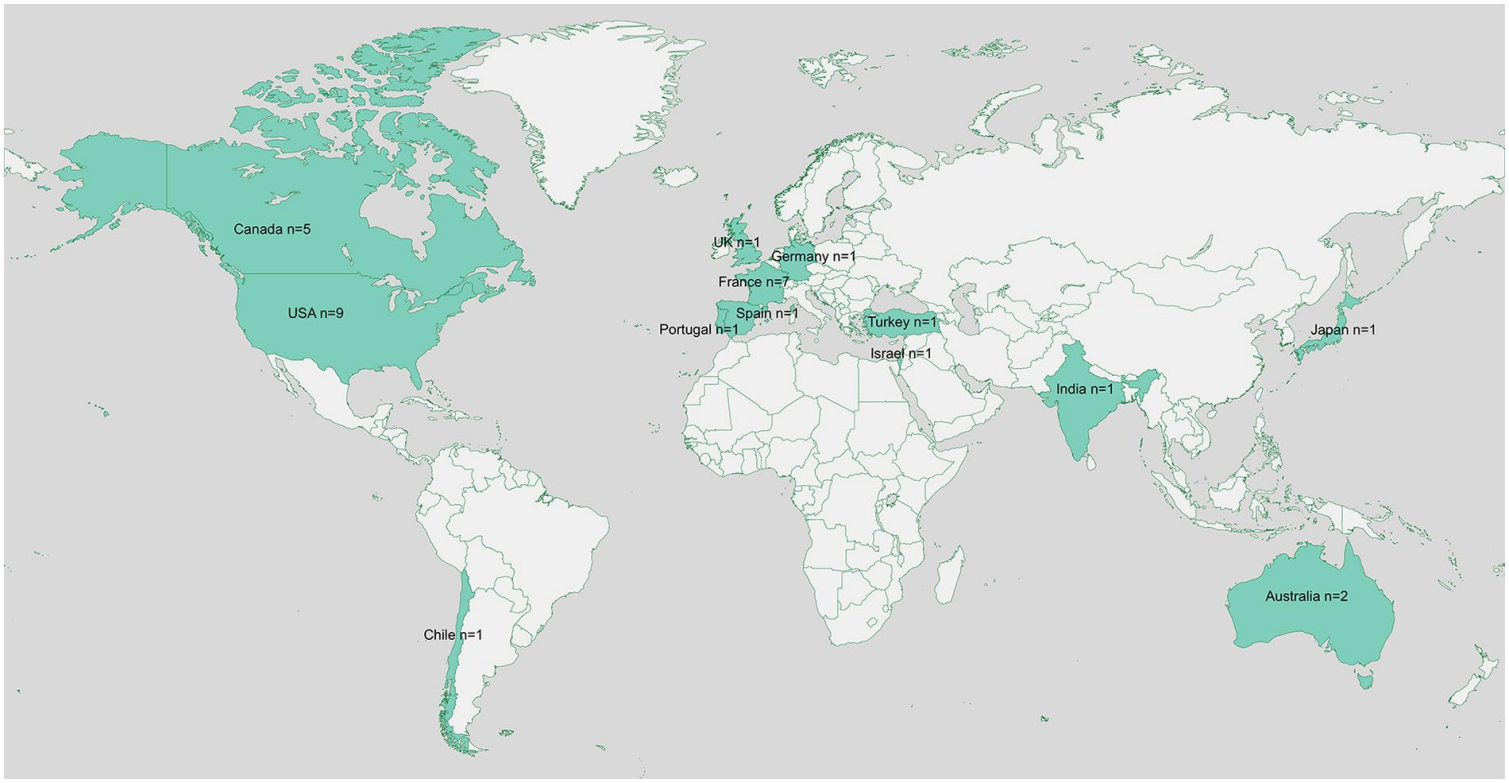
The geographical distribution of reported pediatric cases of intoxication-induced coma. The figure was created with MapChart.

Notably, most articles were published by authors affiliated with hospitals in the USA, Canada and France, in Figure 3, the geographical distribution of the reported cases is shown.

## 4 Discussion

Recent years have seen a rise in accidental exposure of children under the age of 5 to analgesic medications, antipsychotic and antidepressant drugs, and illicit substances resulting in intoxication-induced comas.

When carefully examining the substances involved—excluding paracetamol and ibuprofen, which are the most widely available and accessible medications at home—recent years have shown a notable increase in cannabis intoxication, as reported in the cases included in this work. Cannabis has become increasingly available in edible forms that often resemble appealing food items to children, such as candies, cookies, and baked goods, making them a common cause of accidental ingestion (38). Opioids, on the other hand, are now more accessible due to both a rise in therapeutic prescriptions and an increase in illicit use, especially of synthetic opioids such as fentanyl, with literature reports indicating a marked increase in hospitalizations attributed to opioid poisoning in the paediatric population (5, 39–41).

Most available paediatric protocols for the management of coma in the emergency department setting recommend urine toxicology screening as a second-line investigation, to be performed when the initial clinical assessment fails to identify a specific cause or when there is a well-founded suspicion of poisoning by exogenous substances (1–4).

Intoxication is a relatively more frequent cause of non-traumatic coma, particularly in children younger than 5 years of age, compared to other causes, such as metabolic diseases or diabetic ketoacidosis. Coma due to status epilepticus has also become less frequent than in the past, probably because of changes in pharmacological management and family education (1). On the other hand, it is important to emphasize that infectious causes represent the most frequent forms of coma in absolute terms. In such cases, the need to obtain a urine sample using a sterile technique makes catheterization a useful tool in the management of coma in the Emergency Department—not only for toxicological screening, but also for the assessment of potential urinary tract infections. In general, the literature does not report coma as an indication for performing urinary catheterization (42); however, we believe that the rapidity of execution and sample collection is sufficient to consider it as the method of choice (43).

Despite limitations, rapid urine screening test (RUS) can significantly contribute to a faster identification of neurotoxic agents, particularly in paediatric settings, where accidental ingestion of medications or substances of abuse often occurs unwitnessed or is not reported by caregivers. Urine screening can be completed within minutes and performed in the emergency department, whereas quantitative toxicological analysis requires a dedicated laboratory and may take several hours to days. Therefore, the qualitative toxicology assay can be considered as a point of care testing (POCT) modality, rapidly applicable in the emergency department setting. A high concordance was observed between qualitative and quantitative toxicological analysis in the 14 cases where both were performed, with no false-positive results identified. These findings suggest a reasonable level of reliability of this approach, for detecting the most common substance implicated in intoxication-induced coma. However, the limited number of cases and the lack of larger epidemiological studies may affect the robustness of these information.

In the context of diagnosing and managing coma in an emergency setting, having a quantitative analysis is not essential in initial steps. Moreover, the correlation between plasma and urinary levels and the clinical effect of the toxic substance is not always straightforward, particularly in paediatric patients, who may have different pharmacokinetics compared to adults.

Urine drug screening typically begins with immunoassays, which are widely used because they allow rapid, automated, large-scale testing and are even incorporated into point-of-care and home-testing kits. However, because immunoassays may yield false-positive results and cannot always distinguish between drugs within the same class, their findings are considered preliminary. Confirmatory testing is therefore performed using gas chromatography–mass spectrometry (GC-MS), which is regarded as the gold standard because of its ability to detect minimal quantities and accurately identify specific drugs. Despite its high accuracy and reliability, GC-MS is costly, time-consuming, and requires specialized expertise, so it is generally reserved for confirming positive immunoassay results (3, 44).

Most commercially available tests can identify the substances most frequently involved, including cannabinoids, opioids, benzodiazepines, barbiturates, cocaine, amphetamines, methamphetamines, buprenorphine, and tricyclic antidepressants (3). Therefore, a simple qualitative test may be sufficient to guide the initial decision-making process. Indeed, RUS has relevant limitations that must be carefully considered in clinical interpretation. False-negative results may occur and can reduce the diagnostic sensitivity of qualitative screening tests, particularly for substances that are not routinely included in standard immunoassay panels, such as fentanyl, other synthetic opioids or synthetic cannabinoids. False negatives may occur when the toxic substance is not included in the screening panel, when urine collection occurs outside the optimal detection window, or when urinary concentrations remain below the assay threshold and these characteristics may vary according to the substance involved. False-negative results represent one of the major limitations of rapid urine screening and may have relevant clinical consequences if misinterpreted. A negative screening result may create false reassurance and lead to premature exclusion of intoxication from the differential diagnosis, potentially delaying appropriate treatment. For this reason, RUS should not be used to rule out poisoning. Rather, it should be interpreted as an adjunctive diagnostic tool within the broader clinical context, and further toxicological investigations should be considered whenever clinical suspicion persists despite a negative result. In addition, immunoassay-based tests may be affected by cross-reactivity between structurally related compounds, potentially generating false-positive results (45).

For these reasons, confirmatory quantitative analysis (e.g., GC-MS or LC-MS/MS) remains essential whenever clinically indicated, particularly for positive screening results or when clinical suspicion remains high despite an initial negative result.

However, despite these limitations, RUS may still provide a valuable early diagnostic clue and rapidly orient the diagnostic reasoning in emergency settings, where timely decisions are often required before confirmatory laboratory results become available. In this context, its role is not to establish a definitive diagnosis, but to support early clinical decision-making.

From a practical perspective, severe unexplained coma in children younger than 4 years represents a relatively uncommon but high-stakes clinical presentation. In this selected symptomatic population, where the pre-test probability of toxic ingestion is clinically enriched, the addition of a low-cost qualitative toxicology screen is unlikely to substantially increase overall diagnostic costs and may potentially reduce the use of more invasive or resource-intensive procedures such as lumbar puncture, neuroimaging, or prolonged diagnostic observation. At the same time a timely diagnosis may allow a prompt treatment with an antidote when available and required.

Although not all substances have an available antidote, an early diagnosis can help avoid invasive tests with potential risks of complications. In this review lumbar puncture was performed in 9 patients and was negative in all cases, cranial CT scan was performed in 12 patients and in 6 were unremarkable.

While some guidelines recommend performing urine toxicology screening (3, 4) only in the presence of a highly suggestive history of intoxication, clinical practice indicates that such history is often unreliable. Parents may sometimes withhold information out of fear, thereby compromising the accuracy of the anamnesis. For this reason, physicians should be aware of the importance of including urine toxicology testing as part of the initial evaluation of comatose patients, even in the absence of a witnessed or reported intoxication.

In the published cases included in this review, toxic substances were identified at the initial urine toxicological assessment in 85% of the patients tested. This finding should be interpreted as a descriptive measure of diagnostic yield within the selected published cases and not as an estimate of the diagnostic sensitivity or overall performance of rapid urine screening in the general paediatric emergency population.

Among the 35 positive cases, cannabinoids were the substances most frequently detected, followed by the opioid methadone. Interestingly, in one case methadone positivity was observed only after 36 h, and in another it was found in blood but not in urine, as the standard immunochemical urine screening failed to detect it (28).

Four further cases of urine test negativity were reported. In two cases of clozapine intoxication, initial urine and blood screenings were negative (37); in one of these, clozapine was later quantified in blood, possibly suggesting that the initial screening panel did not include this substance. In another case, the immunoassay was negative, but the authors specified that the assay used did not cover synthetic opioids, such as fentanyl (10). Similarly, in a case of baclofen intoxication, the drug was not included in the screening panel (8). In the case of tea tree oil intoxication, the substance was not identified, as it was not generally routinely screened for toxicologic purposes (12).

The method of collection was not specified in the 41 cases. While urine samples are commonly obtained using a collection bag or by urinary catheterization, in the emergency setting of a comatose child urinary catheterization was the likely method of choice. As a matter of fact, this procedure is associated with low procedural risk, vs. relevant clinical advantages in terms of efficiency and accuracy, and may represent a reasonable and justifiable approach in this context (42). Urine collection methods should be adapted to the clinical setting and the urgency of diagnostic assessment. Non-invasive approaches, such as urine collection bags or spontaneous voiding, may be considered when feasible, although they may be limited by delayed collection and a higher risk of sample contamination. In critically ill children presenting with unexplained coma, where rapid diagnostic orientation may be clinically important, intermittent urinary catheterization may provide a timely and reliable uncontaminated sample. Moreover, urinary catheterization is generally associated more with anxiety than with pain, so that it should be by far and large considered a non traumatic procedure in a comatous child. Specifically, regarding opioids, these substances can cause urinary retention by binding to mu and delta receptors. Therefore, urinary catheterization serves a dual purpose: it facilitates urine sampling for toxicological analysis and provides immediate relief of bladder distension (43).

However, this approach remains invasive, although it may offer clinical advantages in terms of efficiency and sample reliability. Importantly, the risk of urinary tract infection differs between single intermittent catheterization and prolonged indwelling catheterization, with the latter carrying a substantially higher infectious risk. When clinically indicated and performed under appropriate aseptic conditions, catheterization may represent a reasonable option, particularly when urine output monitoring or sterile urine sampling for infectious work-up is also required.

This review has limits. First, the main limitations of this review stem from the heterogeneity of the case reports included as clinical data and treatment details were not consistently or uniformly reported across all cases. Furthermore, the observed mortality rate in our sample (only 1 case) may not accurately reflect the true mortality associated with these intoxications, due to an inherent selection bias: patients who were declared dead on arrival at the emergency department were excluded. Nevertheless, it is important to note that the assessment of mortality falls outside the primary objectives of this review. It should be also remembered that the cases reported in the literature are highly likely to be a minority of all cases encountered in clinical practice due to a report bias and to the limited availability of large international epidemiological surveys. Moreover, the majority of the cases selected in this work showed a drug screening test result, suggesting a potential publication bias favouring studies with positive findings. These biases may have led to an overestimation of the diagnostic performance of RUS.

It should be reminded that reported cases in the literature are highly likely to be a minority of all cases encountered in clinical practice due to a report bias and to the limited availability of large international epidemiological surveys. Most cases were reported by authors based in the USA, Canada, and France; however, this likely reflects greater attention to publishing on this topic in these countries rather than a true geographic distribution of cases.

Finally, the results in these patients were consistent with larger studies conducted in adults (46, 47).

## 5 Conclusion

Toxic ingestion is a recognized cause of coma in the paediatric population. While in adolescents it is often intentional, in infants and young children it is predominantly accidental and may remain unwitnessed or unreported. In many cases, clinical history alone may not be sufficient to guide diagnosis. In this context, performing RUS during the initial emergency assessment—after stabilization of airway and circulation and alongside standard metabolic evaluation—may facilitate earlier diagnostic orientation. The substances most frequently involved in paediatric poisonings are cannabinoids and opioids, which are among the toxic agents most commonly detected by RUS.

Given its low cost, rapid turnaround time, and ease of use, RUS may represent a valuable adjunctive diagnostic tool with a favourable cost–benefit profile. Available data suggest that its early use in selected paediatric patients—particularly children under 4 years of age presenting with unexplained coma or severe alteration of consciousness—may help support timely, targeted management and potentially reduce the need for more invasive investigations. However, RUS should not replace careful clinical assessment, history taking, or standard diagnostic work-up, and its results must always be interpreted within the broader clinical context. In selected cases, urine toxicology testing may therefore be considered earlier in the diagnostic workflow rather than being reserved exclusively as a secondary investigation. Further prospective multicentre studies are needed to better define its diagnostic accuracy and its impact on clinical management.

## Supplementary material

SUPPLEMENTARY TABLE S1 Assessment of the risk of bias in the included studies.

SUPPLEMENTARY TABLE S2 Detailed characteristics of the cases included in the review.
